# Transport and Separation Characteristics of PVDF-Based Nanocomposite Membranes in Membrane Distillation

**DOI:** 10.3390/membranes16040152

**Published:** 2026-04-21

**Authors:** Syed Farzan Ali Shah, Naif A. Darwish, Nabil Abdel Jabbar, Sameer Al-Asheh, Muhammad Qasim, Farouq S. Mjalli

**Affiliations:** 1 Department of Chemical and Biological Engineering, American University of Sharjah, Sharjah 26666, United Arab Emirates; b00102551@aus.edu (S.F.A.S.); ndarwish@aus.edu (N.A.D.); sslasheh@aus.edu (S.A.-A.); mqasim@aus.edu (M.Q.); 2 Materials Science and Engineering Program, College of Arts and Sciences, American University of Sharjah, Sharjah 26666, United Arab Emirates; 3 Petroleum and Chemical Engineering Department, Sultan Qaboos University, Muscat 123, Oman; farouqsm@yahoo.com

**Keywords:** membrane distillation, carbon nano-tubes, phase inversion, PVDF

## Abstract

Water scarcity has increased the need for efficient treatment technologies such as membrane distillation (MD). PMD performance depends strongly on membrane fabrication parameters, particularly polymer concentration and nanoparticle incorporation, which control key transport and separation properties. This study considers fabrication of membranes using different concentrations of polyvinylidene fluoride (PVDF) with the incorporation of different types of nanoparticles to determine the optimum membrane formulation for membrane distillation applications. The results demonstrate that both PVDF concentration and nanoparticle type play a critical role in membrane performance in terms of permeate flux and salt rejection. Among the nanoparticles studied in this work, carbon nanotubes (CNTs) exhibited the most significant enhancement, leading to a substantial increase in water vapor flux while maintaining excellent separation efficiency. The optimized CNT incorporated membrane achieved approximately 99% salt rejection, with superior flux performance, indicating its strong potential for high-efficiency desalination and water treatment using membrane distillation.

## 1. Introduction

Safe and reliable water is essential for health, food production, energy, and economic growth. Only 0.036% of global water is actually accessible for human use, with around 80% of the world’s population facing water supply threats [1]. Increasing population, urbanization and climate change make freshwater resources a challenging issue. In addition, pollution from untreated industrial waste, agricultural runoff, and salty water limit the availability of usable freshwater. These problems make it necessary to develop effective and sustainable water treatment and desalination technologies to provide clean water for the future.

As freshwater resources become increasingly limited, seawater offers a promising alternative due to its abundance. Water covers over 70% of Earth’s surface, yet about 97.5% of it is salty water [2]. Through modern desalination technologies, seawater can be converted into safe and potable water, providing a reliable supply for drinking, industrial, and agricultural needs. Utilizing seawater not only helps address water scarcity but also reduces the pressure on natural freshwater sources, making it an important component for management and strategies of sustainable water.

There are several technologies available for treating seawater; these are generally categorized as thermal-based or membrane-based technologies. However, membrane-based technologies are considered the most efficient and widely used due to their lower energy consumption, high separation efficiency, and ability to produce high-quality water with minimal chemical use [3,4,5]. Among these, processes such as reverse osmosis, nanofiltration, and membrane distillation depend on selective separation through semi-permeable membranes. In a membrane-based processes, water molecules transport through the membrane while salts and other impurities are retained, allowing for effective purification. The working principle typically depends on either pressure-driven flow, as in reverse osmosis, or thermally driven vapor transport, as in membrane distillation (MD), where a hydrophobic membrane allows only water vapor to pass while rejecting liquid water and dissolved salts [6,7].

MD technology has gained significant attention in recent years as a promising method for seawater desalination and wastewater treatment. Unlike traditional pressure-driven membrane processes, MD operates at lower pressures and moderate temperatures. The temperature difference across the MD porous and hydrophobic membrane creates a vapor pressure difference, which results in the transfer of volatile components from the hot feed side through the membrane pores as vapor, which is then condensate on the permeate side. The other dissolved components are prevented from passing through the membrane and are thus excluded by the membrane [8]. Operating at low temperature and pressure makes it energy-efficient process, especially when integrated with renewable heat sources such as solar or waste heat. This would offer high salt rejection and excellent water quality, even when treating highly saline or contaminated water that makes it challenging compared to other conventional technologies [9]. Furthermore, MD membranes are less prone to fouling and scaling compared to pressure-driven membranes, which extends membrane life and reduces operational costs. These advantages make MD a versatile and sustainable solution for producing clean water in regions facing severe water scarcity or limited access to freshwater resources. Pressure-driven membranes like RO are the most widely used method globally. This is because RO membranes attain more water flux and have a low initial cost compared to other membrane desalination techniques [10]. Moreover, RO membrane modules are readily available commercially and can be easily implemented in small-scale applications, which further contributes to their widespread use.

The performance of the MD system largely depends on the quality and properties of the membrane used. A high-performing membrane is essential for achieving maximum water flux, high salt rejection, and long-term stability [11]. Key characteristics such as porosity, pore size, thickness, and surface hydrophobicity play a critical role in allowing efficient water vapor transport while preventing wetting and fouling. Additionally, mechanical strength and thermal stability are important characteristics that ensure that the membrane can operate reliably under different conditions [12]. Therefore, developing or selecting the best-performing membrane is crucial for optimizing MD efficiency, reducing energy consumption, and maintaining consistent water production over time.

The concentration of the polymer plays a vital role in controlling membrane performance, as it strongly affects solution viscosity, phase separation behaviour, and polymer–solvent–non-solvent interactions during membrane formation. At lower polymer concentrations, rapid mixing can be achieved, leading to the formation of highly porous structures with larger pores and finger-like macro-voids. In contrast, increasing the polymer concentration results in delayed phase separation, producing denser membranes with smaller pore sizes and thus reduced porosity and improved mechanical strength [13,14]. Therefore, careful selection of polymer concentration is essential to tailor membrane morphology and achieve a desirable balance between permeability, selectivity, and structural stability.

In addition to polymer concentration, the incorporation of nanoparticles into membranes has a significant impact on their performance. Nanoparticles can enhance such key properties as porosity, surface roughness, hydrophobicity, and mechanical strength, which are critical for efficient membrane distillation process [15]. Nanoparticles enhance water vapor transport and reduce membrane wetting, thereby increasing water flux while maintaining high salt rejection [16]. Different types of nanoparticles, such as carbon nanotubes, silica, or metal oxides, can be tailored to improve membrane properties. Therefore, the addition of nanoparticles is a promising strategy with which to optimize membrane performance and achieve higher efficiency and durability in MD systems.

Nanoparticles like CNTs are made by rolling sheets of tiny tube-shaped graphite material. CNTs can be made by three methods: arc discharge, laser ablation, and chemical vapor deposition (CVD) [17,18,19]. Among them CVD is mostly used for CNTs. However, it relies on non-renewable carbon sources like methane and acetylene, along with metal catalysts [19]. The process generates harmful emissions, contributing to environmental pollution and sustainability concerns. However, this environmental impact can be solved by using biomass raw material and by using a green catalyst. CNTs have a few problems such as their toxicity to living cells and low bioavailability [20], but not all CNTs are harmful; their toxicity depends on their size and shape. Generally, longer and thicker CNTs are more harmful than shorter and smaller ones [21].

Membranes can be fabricated by different techniques. Common methods used to fabricate membrane include phase inversion, interfacial polymerization, stretching, track etching and electrospinning [22]. Among these various membrane fabrication techniques, non-solvent induced phase separation (NIPS) is considered as one of the simplest and most widely used methods. In this technique, a polymer, such as PVDF, is dissolved in a suitable solvent to form a homogeneous solution, which is then cast into a thin film and immersed in a non-solvent bath, typically water [23]. The interaction between the solvent and non-solvent induces polymer precipitation, resulting in the formation of a porous membrane structure. NIPS is widely used due to its simplicity, cost-effectiveness, and versatility, as it allows precise control over key membrane characteristics such as pore size, porosity, and thickness. These characteristics are typically achieved by variations in polymer concentration, solvent selection, and coagulation conditions, thus making NIPS an effective fabrication method for producing membranes tailored for membrane distillation and other water treatment applications [24,25].

This study considers development of membranes for MD using different polymer concentrations. The optimum membrane was selected based on water flux and salt rejection performance during saline water treatment using direct contact membrane distillation (DCMD) technology. To improve membrane flux, a modified membrane was also developed by incorporating carbon nanotubes (CNTs), graphene oxide (GO), and chitosan as nanofillers. Furthermore, various CNT loadings were examined to identify the optimum CNT concentration that yields the best membrane performance. This work aims to develop a membrane with CNT nanofillers for membrane distillation, with the goal of increasing water flux compared to conventional membranes.

## 2. Materials and Methods

All experimental measurements were performed at least twice, and the errors were determined from standard error (SE) method. Temperature was measured using a digital thermocouple, provided by Gain Express, Hong Kong, Hong Kong (S.A.R) with an accuracy of ±0.1 °C, while the flow rates were controlled by a peristaltic pumps provided by Kamoer, Shanghai, China. Mass measurements were carried out using an analytical balance provided by WANT Balance Instrument CO., Ltd., Jiangsu, China with a readability of ±0.1 g.

### 2.1. Materials

All chemicals used in this work were of analytical grade and used without further purification. Polyvinyl diflourides (PVDF MW 534,000) as polymer, Multiwalled carbon nano-tubes (50–90 nm diameter, 95% carbon basis), graphene oxide (0.5–10 μm size, industrial grade, 97% purity), and chitosan (low MW) were supplied by Sigma-Aldrich, (St Quentin Fallavier, France). N-Methyl-2-pyrrolidone (extra pure AR, 99%) was supplied by Sisco Research Laboratories, Mumbai, India. Sodium chloride (Hi-AR ASSAY 98.5–102%) was supplied by HiMedia Laboratories, Mumbai, India.

### 2.2. Membrane Preparation

#### 2.2.1. Preparation of PVDF Solution

The hydrophobic flat-sheet membranes were prepared. In case of nano-particles present, they were first dispersed in N-Methyl-2-pyrrolidone (NMP) for 1 h using ultrasonication until formation of uniform dispersion. After ultrasonication, PVDF was gradually added to the solvent while continuously heating and stirring for 6 h at 50 °C. The resulting dope solution was left undisturbed to allow removal of any trapped air molecules, and the solution was allowed to cool to room temperature.

#### 2.2.2. Preparation of Flat Sheet Membrane Using Phase Inversion

Polymeric membranes are typically fabricated using the phase inversion process. A typical phase inversion fabrication setup consists of a precision casting head or doctor-blade assembly, a flat and level casting table, and moving rod. In this process, a polymer solution changes from a liquid phase to a solid phase. Before the formation of the solid phase, a liquid–liquid separation takes place, causing the polymer solution to split into two phases: one with a higher polymer content and the other with less polymer content. The phase with more polymer content can then be solidified to form the membrane structure using gelation or crystallization [26]. The polymer-poor phase creates pores in the solid membrane structure. The process known as immersion precipitation, or non-solvent induced phase separation (NIPS), requires placing the polymer solution into a non-solvent bath. For this process, the polymer must be soluble in a solvent or solvent mixture, allowing a variety of polymers to be used in membrane synthesis.

The homogeneous polymer solution was poured onto a substrate, such as a glass plate or polymeric support, and spread with a casting knife to create a uniform layer. Each membrane had a thickness of around 150 μm controlled by an adjustable casting knife. This setup enables precise control over film formation, ensuring consistent thickness and a reproducible surface structure. After that, the glass plate is immediately dipped into a coagulation bath, where the solvent and non-solvent start to exchange. The interaction between NMP and water also affects the final morphology of the membrane. Moreover, the temperature of the water in the coagulation bath also affects the final structure of the membrane [27]. The choice of solvent is dependent upon the polymer [25]. Aprotic solvents like NMP have high chemical affinity for polymers. In NIPS, the non-solvent such as water should have a strong affinity for the solvent while having little or no affinity for the polymer. As the polymer and solvent solution is dipped into the coagulation bath, the non-solvent water diffuses into the membrane solution while the NMP starts to diffuse into the non-solvent. Since non-solvent water has a low affinity for polymers, it become insoluble. This results in phase separation of the membrane, with the polymer-rich surface solidifying to make a membrane matrix, while the polymer-poor phase forms pores. The fast exchange between the solvent and non-solvent results in finger-like pores, while delayed exchange forms sponge-like pores [23]. After some time, the membrane solidifies and floats to the top of the bath. The programmable film coater used for the phase inversion process was supplied by TMAXCN, Xiamen, China.

### 2.3. Membrane Characterization

#### 2.3.1. Membrane Morphology

The morphology of the membrane was examined using scanning electron microscopy, or SEM (FE-SEM, TESCAN-MAGNA, Brno, Czech Republic). Prior to SEM analysis, the membrane samples were attached to a sample holder by carbon tape. The samples were then coated with a thin layer of gold using a sputter-coating (SC7620, Quorum, East Sussex UK) machine to enhance its electronic conductivity during sample analysis. The morphology of the membrane was observed based on the SEM imaging using ImageJ (Imagej.Net 1.54g) software.

Morphology provides information about images that reveal the dense skin layer and changes in a membrane’s porosity at different polymer concentrations [28,29]. SEM can confirm the successful incorporation of additives into the membrane structure, such as CNTs or graphene oxide in polyamide layers, enhancing hydrophobic properties and water permeability [30].

#### 2.3.2. Hydrophobicity/Hydrophilicity

The hydrophobicity/hydrophilicity of the membrane was measured using a contact angle goniometer device (DSA100M, Kruss GmbH, Hamburg, Germany). In this case, two membrane samples were attached to a glass sheet and placed on the testing plate. A 5 μL droplet was placed on the surface of the membrane, and the contact angle was then measured.

#### 2.3.3. Mean Pore Size Calculation

The average pore size of the developed membranes was evaluated using SEM images processed in ImageJ software (Imagej.Net 1.54g). Pore diameters were measured at several randomly selected regions on each membrane surface to obtain representative values, and the mean pore size was calculated. This method allowed for a reliable assessment of membrane morphology and its relevance to separation performance [31].

#### 2.3.4. Porosity

The membrane porosity, *ε*, was determined by the gravitational method, which is based on the ratio of the pore volume to the net volume of the porous membrane [32,33]. For each membrane sample, the dry membrane was first weighed (*m_d_*), followed by the immersion of the membrane in isopropanol for 2 h. According to this method, the porosity can be calculated using the following equation:

(1) $$\varepsilon \;=\;\frac{\frac{m_{w}\;-\;m_{d}}{\rho_{i}}}{\frac{m_{w}\;-\;m_{d}}{\rho_{i}}\;+\;\frac{m_{d}}{\rho_{p}}\;}\;\times \;100$$

where m_w_ and m_d_ are the mass of wet and dry membrane (kg), respectively; $\rho_{i}$ is the density of isopropanol; and $\rho_{p}$ is the density of the polymer. The numerator of the above equation represents the pore volume, while the denominator represents the net membrane volume. This calculation was carried out because isopropanol readily enters and fills the pores, allowing the difference between dry and wet weight with a known density to be used to accurately calculate porosity [34]. It should be noted that porosity was calculated assuming negligible swelling during the isopropanol gravimetric measurement; therefore, the values are mainly used for relative comparison among the fabricated membranes.

#### 2.3.5. Water Flux

A lab-scale DCMD setup was used to evaluate the desalination performance using the membranes developed in this work. A piece of the membrane was placed between two pads in a module. The feed solution (salty water 35 g/mL) at a high temperature of 60 °C and the low-temperature permeate solution (DI water at 20–25 °C) were circulated using two peristaltic pumps. The test was conducted for a period of around 2 h. The amount of water that overflowed (Δ*m*), in kg, from the permeate side was measured after a specific time interval (Δ*t*) to calculate the flux using the following equation:

(2) $$J_{W}\;=\;\frac{\Delta m}{A\;\times \;\Delta t}$$

where *A* is the cross-sectional area of membrane in m^2^.

#### 2.3.6. Salt Rejection

The salt rejection efficiency of the membranes was evaluated by measuring the salt concentration in the feed and permeate solutions, using saline wastewater of known initial concentration as the feed. The salt rejection in both streams was measured by the TDS analyser (HI2003-edge, HANNA, Cluj, Romania). The salt rejection (*R*) percentage was calculated using the following equation [35]:

(3) $$R\;=\;\left(1\;-\;\frac{C_{p}}{C_{f}}\right)\;\times \;100$$

### 2.4. Direct Contact Membrane Distillation Setup

**Figure 1:**
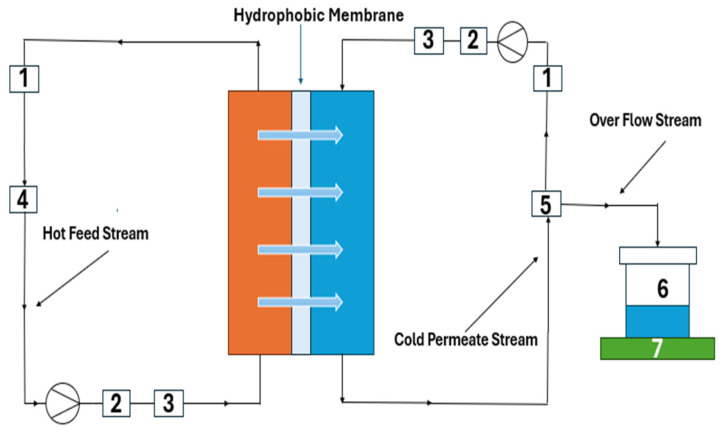
Schematic diagram of the DCMD setup: (1) conductivity meter; (2) flow transmitter; (3) temperature transmitter; (4) heater; (5) cooler; (6) product water collection tank; (7) balance.

A schematic of the experimental direct contact membrane distillation (DCMD) setup used in this study is shown in Figure 1. In this setup, a flat-sheet membrane module made of acrylic with an effective area of 0.0021 m^2^ was used. A fresh membrane was used for each experiment. The feed and permeate streams were pumped in a co-current pattern using two peristaltic pumps. The feed temperature and flow rates were varied for each run, while the permeate temperature and flow rates were maintained at about 22 °C and 100 mL/min, respectively, throughout all experiments. The temperature of the permeate side was controlled using an external condenser. Initially, deionized (DI) water was filled into the permeate side graduated tank, which was placed on an electronic balance with an accuracy of 0.1 g. The mass of water transferred was determined from the difference between initial and final readings of the balance. A TDS meter was used to measure the solution’s salt rejection in the permeate tank. The hot feed solution was initially placed in a feed tank stirred using a magnetic stirrer. Temperature probes with an accuracy of ±0.1 °C were used to monitor the temperatures of both streams at the inlets and outlets of the membrane module.

## 3. Results and Discussion

### 3.1. PVDF Membrane Characterization

#### 3.1.1. SEM Images and Pore Size Distribution

**Figure 2:**
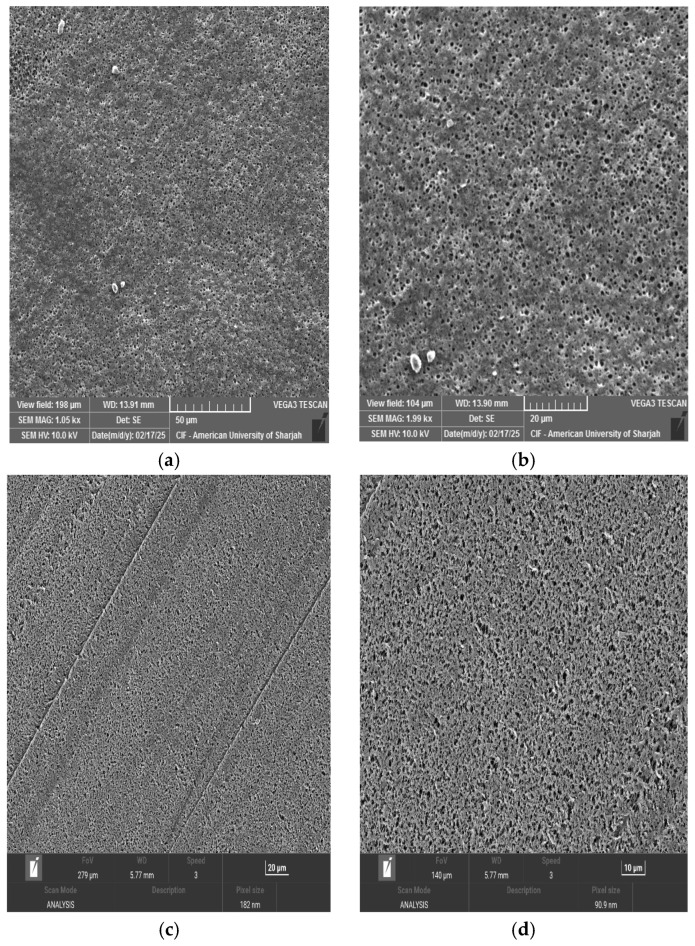
SEM images of the membrane at different PVDF concentrations and different magnifications: (**a**) 13 wt% PVDF at 50 μm; (**b**) 13 wt% PVDF at 20 μm; (**c**) 20 wt% PVDF at 20 μm; (**d**) 20 wt% PVDF at 10 μm.

PVDF membranes prepared under identical fabrication conditions but with different polymer concentrations, namely 13 wt% and 20 wt%, exhibited distinct surface morphologies. The SEM images are shown in Figure 2 at different magnifications. At 20 μm magnification, the 20 wt% PVDF membrane cast reveals a denser surface (Figure 2c) with reduced pore size, while the 13 wt% PVDF membrane (Figure 2b) reveals a more open and porous structure. This difference can be attributed to the increase in polymer content that enhances dope viscosity, thus reducing the rate of solvent–non-solvent exchange during phase inversion [36,37]. Consequently, higher polymer loading results in delayed demixing and formation of a compact surface layer.

**Figure 3:**
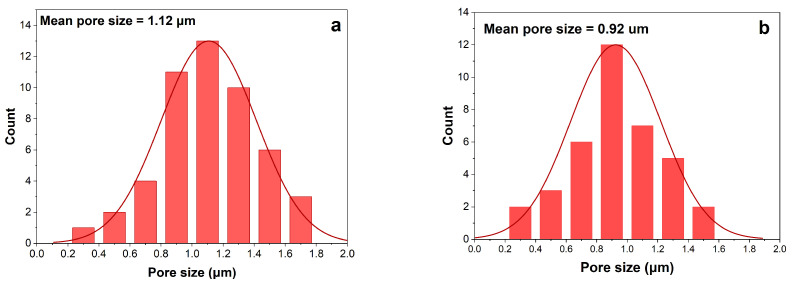
Pore size distribution of membrane at different PVDF concentration: (**a**) 13 wt% PVDF; (**b**) 20 wt% PVDF.

The pore size distribution is shown in Figure 3. It can be said that both membranes show almost equally pore size distribution. However, the average pore size of the 20 wt% PVDF membrane was decreased compared to that of the 13 wt%. This is because the polymer-rich phase dominates earlier during phase separation, limiting the growth of polymer-lean (pore-forming) regions [38]. At the same time, the pore formation kinetics can be dominated by diffusion-limited processes rather than rapid demixing, which tends to stabilize the pore size distribution. This indicates that the pore size might decrease further, only slowly, and the variation in pore diameters narrows [39].

**Figure 4:**
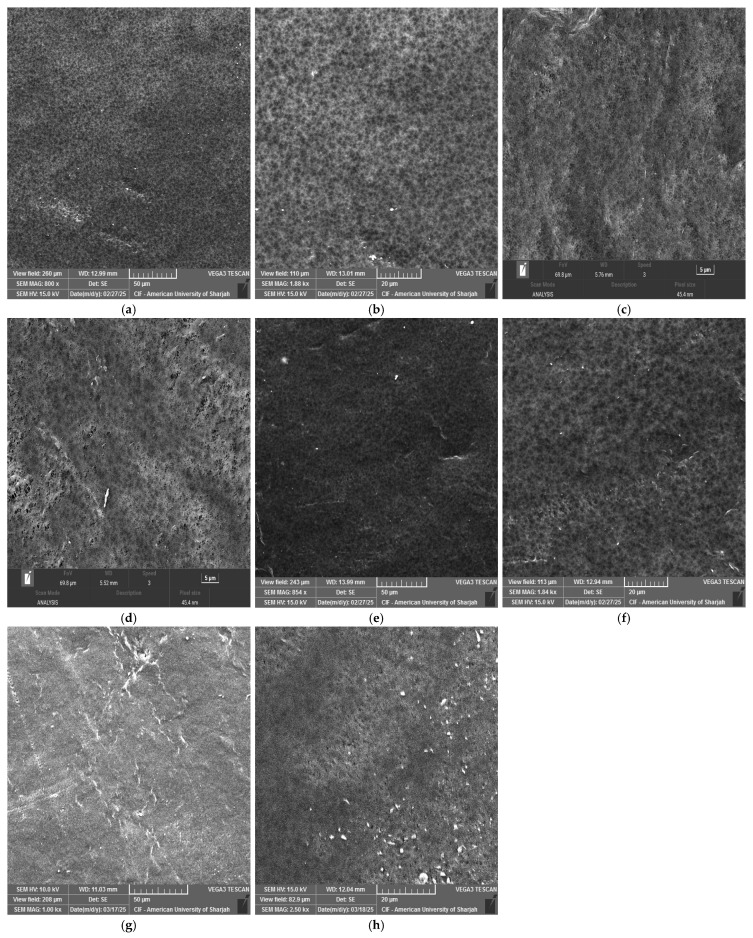
SEM images of the membranes with different additives: (**a**) PVDF-0.5 wt% CNT at 50 μm; (**b**) PVDF-0.5 wt% CNT at 20 μm; (**c**) PVDF-0.7 wt% CNT at 5 μm; (**d**) PVDF-0.7 wt% CNT at 5 μm; (**e**) PVDF-0.5 wt% GO at 50 μm; (**f**) PVDF-0.5 wt% GO at 20 μm; (**g**) PVDF-0.5 wt% CH at 50 μm; (**h**) PVDF-0.5 wt% CH at 20 μm.

The SEM images revealed that the pores appear blurred or partially covered upon adding nanoparticles in the membranes (Figure 4). The SEM images for 13% PVDF-0.5 wt% CNT and 13% PVDF-0.7 wt% CNT images captured at 20 μm and 5 μm, respectively, showed that increasing CNT concentration causes the pores to be blurred or partially covered (Figure 4b,c). This is mainly due to the interaction between the CNT additives and the PVDF matrix, which alters the solvent–non-solvent exchange rate and increases solution viscosity. For CNT particles, it is expected that their high surface area and strong polymer interaction reduce demixing in localized regions, resulting in a smoother and denser appearance [40].

Figure 4e displays the morphology of the membrane containing 0.5 wt% GO. Relative to CNTs, the membrane shows a denser top layer due to the two-dimensional nanosheet structure and high specific surface area of GO. At low concentrations, these sheets can overlap, creating a structure that is highly extended relative to its mass. It has been reported that GO increases the accelerated phase change, producing a thin but compact top layer [41].

The images presented in Figure 4g and h show that chitosan was poorly dispersed in the PVDF–NMP casting solution. This is due to the inherently hydrophilic nature of chitosan, whereas PVDF is strongly hydrophobic, which limits its miscibility in a common casting solution. Poor compatibility can lead to aggregation, resulting in a visible surface of chitosan particles.

**Figure 5:**
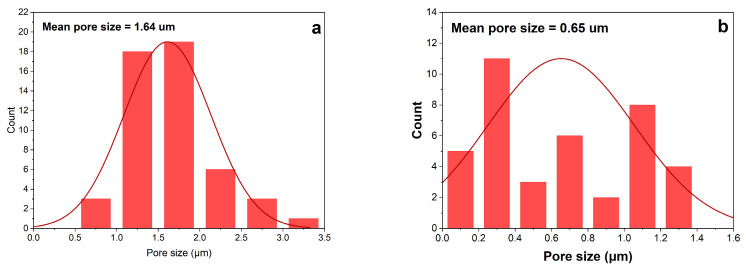
Pore size distribution of membrane at different concentrations of CNT; (**a**) PVDF-0.5 wt% CNT; (**b**) PVDF-0.7 wt% CNT.

Pore size distributions of 0.5 wt% CNT and 0.7 wt% CNT are shown in Figure 5. The 0.5 wt% CNT membrane shows a more uniform pore size distribution than the 0.7 wt% CNT. Although the difference was very small, the multiwalled nature of CNTs made the blending process more difficult, resulting in an uneven pore distribution in the membrane [35,42]. Figure 4d shows that some areas feature larger pores, while others remain denser, resulting in a non-uniform pore structure.

#### 3.1.2. Porosity Test Results

**Figure 6:**
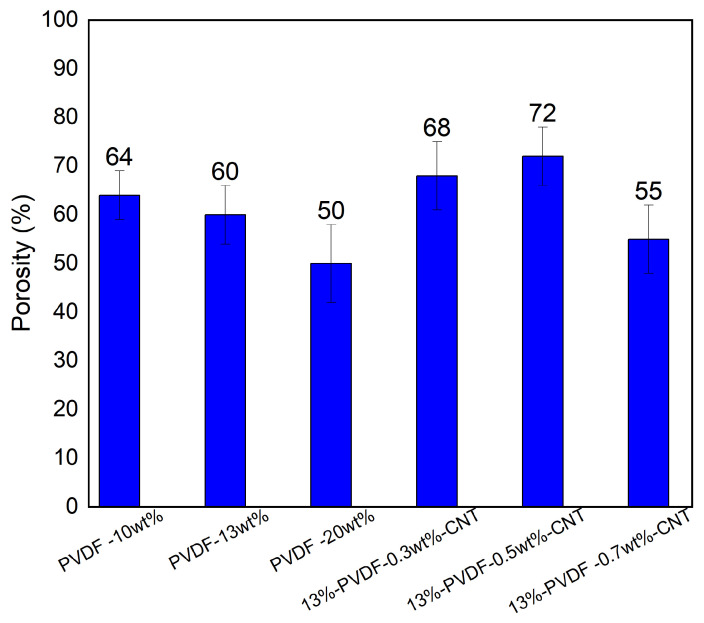
Porosity results for synthesized PVDF membranes at different concentrations and different concentrations of CNT nanoparticles.

The porosity of the fabricated membranes plays a crucial role in determining mass transfer performance and flux behaviour during membrane distillation processes. The porosity results are presented in Figure 6 for the different types of membranes fabricated in this work, where bars show the average values of replicates. The pristine PVDF membrane showed that porosity decreases with increasing PVDF concentration; membrane porosity decreased from 64% to 50% as PVDF concentration increased from 10% to 20%. This decrease in porosity is attributed to the higher polymer concentration that reduces the solvent–non-solvent exchange and thus limits polymer chain mobility, which suppresses phase separation and pore formation. One study on polysulfone membranes demonstrated that higher polymer concentrations resulted in denser structures with lower void fraction and reduced water permeation [33].

Membrane porosity is strongly influenced by several parameters, such as coagulation bath temperature and polymer molecular weight [43]. Moreover, the solvent’s affinity for both the non-solvent and the polymer strongly governs pore formation; highly miscible solvents (e.g., DMF) promote faster demixing and higher porosity, whereas low-compatibility solvents (e.g., NMP) yield denser membranes [22].

The incorporation of a small amount of CNTs (0.3% to 0.7%) into the 13% PVDF matrix led to an increase in membrane porosity (Figure 6), which can be attributed to the enhanced phase separation during the non-solvent induced phase inversion process. It has been reported that CNT can act as a nucleation site within the polymer matrix, promoting faster demixing between the solvent and non-solvent phases and thereby creating more voids and higher overall porosity [33].

At higher CNT concentrations, the porosity of the PVDF membrane does not increase further. This behaviour is likely due to a significant increase in the viscosity of the dope solution, which suppresses the solvent–non-solvent exchange rate during the phase inversion process. Excess CNTs tend to agglomerate because of strong van der Waals interactions, forming clusters that block pore channels and hinder uniform pore formation [44]. At elevated CNT concentrations, aggregation occurs, which slows the demixing process and leads to a denser membrane structure with reduced void volume. Among the tested membranes, 13% PVDF-0.5 wt% CNT can be considered the optimum, as it exhibits the highest porosity, provides a more favorable vapor transport pathway, and consequently achieves higher flux.

#### 3.1.3. Contact Angle Analysis

The contact angle reflects membrane surface wettability, which directly influences the success of the membrane distillation process. A higher contact angle corresponds to a more hydrophobic surface, which is desirable in processes involving thermal desalination, as it helps prevent pore wetting and maintains stable flux.

**Figure 7:**
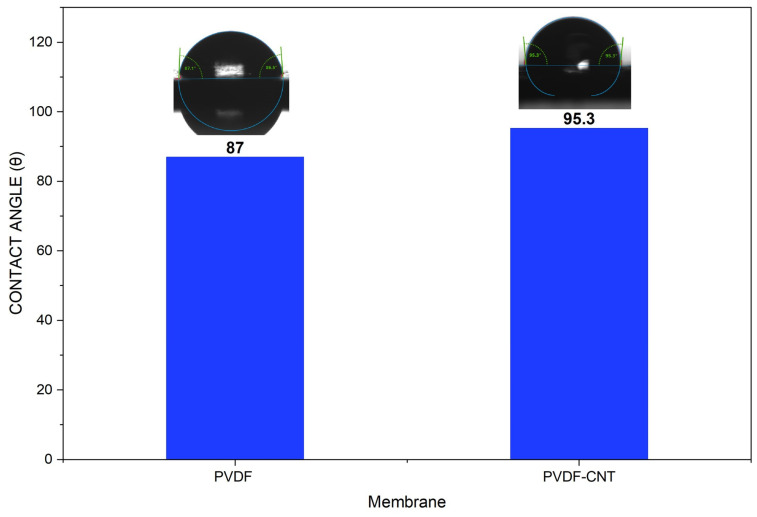
Contact angle for PVDF and PVDF-CNT membranes.

Figure 7 shows the contact angle of the 13% PVDF and 13% PVDF-0.5 wt% CNT membranes developed in this work. The incorporation of carbon nanotubes (CNTs) into the membrane matrix significantly increases the water contact angle from 87° to 95.3°, indicating enhanced membrane hydrophobicity. This improvement is attributed to the inherent hydrophobic nature of CNTs and their ability to modify surface roughness and surface energy when well dispersed within the polymer matrix. Increased hydrophobicity is particularly beneficial for membrane distillation, as it improves resistance to membrane wettability and enhances long-term operational stability [45,46].

#### 3.1.4. FTIR Results

**Figure 8:**
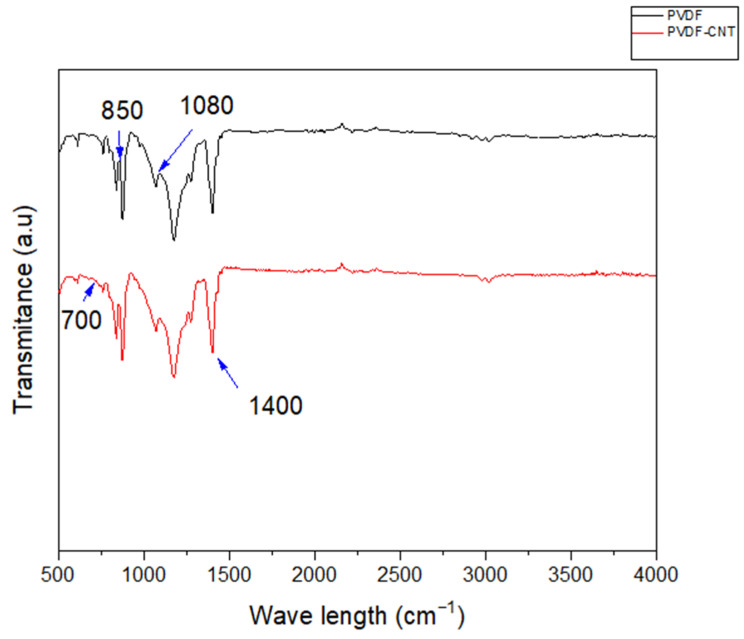
FTIR spectra for 13% PVDF and 13% PVDF-CNT-0.5% membranes.

Fourier transform infrared spectroscopy (FTIR) spectra (Thermo Scientific Id7 ATR) can identify the functional groups and chemical bonds present in a material. Figure 8 shows peaks between 500 and 980 cm^−1^, indicating the presence of both α (major) and β-phases (minor) of PVDF [47]. The peaks observed between 850 and 900 cm^−1^ can be attributed to the- β-phase, and the remaining peaks represent the α-phase [48,49]. Moreover, the peak around 1234 cm^−1^ reveals the presence of the β-phase [47]. The peak around 1400 cm^−1^ represents the presence of CH_2_ [50]. In the pristine PVDF membrane, characteristic absorption peaks were observed at around 1070–1080 cm^−1^ and 840–880 cm^−1^, corresponding to the C–F stretching and C–H bending vibrations of the PVDF backbone.

After incorporating CNTs into the PVDF, the FTIR signals slightly changed, with a minor decrease in the intensity and a change of positions for the wavenumbers between 700 and 860 cm^−1^, respectively. Since CNTs were present at very low concentrations relative to PVDF during the membrane fabrication, the signals corresponding to C-C, C-O, and C-H are either very weak or not detectable [51,52].

#### 3.1.5. Thermogravimetric Analysis

**Figure 9:**
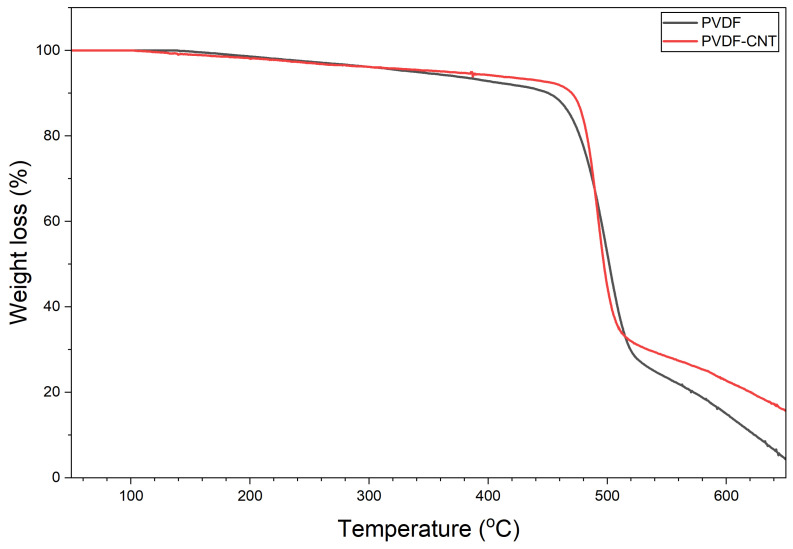
TGA analysis for 13% PVDF and 13% PVDF-CNT-0.5% membranes.

Thermogravimetric analysis (TGA) was performed in the presence of nitrogen using STA 6000 Perkin Elmer Thermal Analyzer, Waltham, Massachusetts, US. The temperature range for this analysis was set between 50 °C and 650 °C. Figure 9 shows the TGA results for the PVDF and PVDF-CNT membranes fabricated in this work. A major weight loss in the PVDF membrane was observed at around 420 °C, which corresponds to the breakdown of the –CH_2_–CF_2_– chains within the polymer backbone [53]. The PVDF polymer backbone remains thermally stable up to ~420 °C, which indicates that decomposition of the polymer does not occur below this temperature.

Decomposition of PVDF–CNT systems shifts to slightly higher temperatures, usually around 450 °C. This improvement is attributed to the strong interfacial interaction between the PVDF chains and the CNT surfaces, which restrict chain mobility and delay thermal degradation [54]. Additionally, the residual mass (char yield) is significant at higher temperatures of 550 °C as compared to pristine PVDF. This indicates that CNT promotes the formation of thermally stable carbonaceous structures that resist decomposition at elevated temperatures and enhance interfacial interactions, thereby reducing the degradation of PVDF chains. Additionally, CNT can act as a physical barrier, limiting polymer chain mobility and further promoting these thermally stable structures [41].

In membrane distillation, even though membranes typically operate below 90 °C, TGA is performed to evaluate the thermal stability of the membrane material itself. The TGA determines the temperature at which the polymer starts to degrade. This ensures that the membrane can safely withstand long-term heating, thermal cycling, and unexpected temperature fluctuations during operation.

### 3.2. Membrane Performance

#### 3.2.1. Effect of Polymer Concentration on Flux and Rejection

The PVDF membranes were successfully fabricated using three different polymer concentrations: 10, 13, and 20 wt%. Each membrane was evaluated using a laboratory-scale direct contact membrane distillation (DCMD) cell with saline feed solution at 60 °C feed side temperature to assess flux and salt rejection performance. The experimental results showed distinct differences in performance depending on the PVDF concentration (Table 1).

The results shown in Table 1 indicate that among the membranes tested, the membrane prepared using 13 wt% PVDF demonstrated the most balanced overall performance. It resulted in high water flux of 8.65 LMH together with excellent salt rejection. The 13 wt% PVDF resulted in an optimal pore structure, providing sufficient porosity for vapor transport while maintaining structural stability and adequate hydrophobicity to prevent pore wetting.

The membrane fabricated using 10 wt% PVDF showed higher flux of 9.2 LMH compared to that of 13 wt%. Due to the lower polymer concentration, the resulting structure was highly porous and mechanically weaker, leading to larger pore size and an increased risk of pore wetting [55]. Although this membrane exhibited relatively high initial flux, its salt rejection was lower, indicating that excessive porosity reduced the selective barrier properties required for effective MD operation. In contrast, the membrane prepared using 20 wt% PVDF demonstrated lower flux of 6.5 LMH with 99% salt rejection. The higher polymer concentration revealed a denser structure with lower porosity and small pore ore size, which restricts vapor transport across the membrane.

The results presented in Table 1 emphasize that polymer concentration strongly influences membrane morphology and performance. Low PVDF concentrations may enhance permeability but compromise mechanical strength and durability, whereas higher concentrations improve structural stability at the cost of reduced flux. Thus, the 13 wt% PVDF membrane can be considered an optimal balance between permeability, selectivity, and mechanical strength for DCMD applications. This is consistent with a previous study [43], which demonstrated that increasing polymer concentration in the dope solution results in a decrease in the flux [56]. This was attributed to the formation of a thicker and denser outer skin layer, underscoring the importance of optimizing polymer content for long-term membrane performance.

#### 3.2.2. Effect of Nanoparticles on Flux and Rejection

Three 13% PVDF membranes were fabricated by incorporating different additives of 0.5% each—carbon nanotubes (CNTs), graphene oxide (GO), and chitosan—into the polymer matrix. These additives are expected to enhance the structural and transport properties of the membranes. The three fabricated membranes were tested in a direct contact membrane distillation (DCMD) cell under similar conditions and with saline feed solution to evaluate water flux and salt rejection. The results (Table 2) showed notable differences in flux depending on the additive used.

The CNT-modified membrane achieved the highest water flux of 10.5 LMH compared to the other samples. CNTs facilitated the formation of well-connected pore channels due to their one-dimensional tubular structure and improved the hydrophobic nature of the membrane [57]. This combination allowed efficient vapor transport while maintaining stability against wetting. Compared to the membrane without carbon nanotube (Table 1), this result showed a 21.4% increase in flux, while salt rejection remained at 99%.

The GO-modified membrane demonstrated a performance very close to that of the CNT membrane with a slightly lower flux, 10 LMH. The layered structure and oxygen-containing functional groups of GO may have contributed to the improved dispersion in the polymer matrix and enhanced surface roughness, which aided water transport. However, compared to CNT, GO showed a larger error in the measurements. This could be attributed to the manner in which the GO was dispersed. GO formed of pristine two-dimensional sheets exhibited a strong tendency to aggregate due to their large planar surface and van der Waals interactions, requiring longer times to achieve a reasonably uniform dispersion. Ultrasonic treatment can also cause fragmentation, defects, and changes in GO structure. Fragmented or defected GO can reduce uniformity in the membrane, leading to variations in flux measurements [58]. In contrast, CNTs, with their one-dimensional tubular structure, dispersed relatively more easily in NMP, as their smaller lateral dimension reduces restacking. Another investigation [59] reported that GO membranes have lower pore density and surface roughness, resulting in compromised performance compared to CNT membranes.

The chitosan-modified membrane showed the lowest flux among the three formulations, as illustrated in Table 2. The hydrophilic nature of chitosan caused partial pore wetting and reduced vapor transport efficiency, thereby limiting the overall membrane performance [60]. The error in the flux measurements could be attributed to differences in chitosan dispersion and dissolution behaviour. Chitosan is inherently hydrophilic and dissolves only in acidic aqueous solvents, such as dilute acetic acid [58,61], through protonation of its amino groups, while remaining insoluble in aprotic solvents like NMP. Ultrasonication can aid dispersion or accelerate dissolution in suitable solvents by breaking up aggregates and improving mixing; however, excessive sonication to achieve complete dispersion can cause polymer chain breakage, reduced molecular weight, and structural disorder [61]. These variations in solubility, dispersion uniformity, and potential polymer degradation result in a non-uniform membrane structure, leading to significant fluctuations in the measured flux. Moreover, GO and chitosan contain hydrophilic functional groups which can reduce surface hydrophobicity, whereas CNTs enhance hydrophobicity due to their non-polar carbon structure.

#### 3.2.3. Effect of CNT Concentration on Flux and Rejection

Carbon-based additives were employed in this study since these additives are known to enhance membrane performance and are also known for their chemical stability and compatibility with PVDF polymer matrix. The concentrations of carbon additives were carefully chosen to maximize their loading in the membrane matrix while maintaining suitable rheological properties for membrane casting. This was to ensure that the casting solution remained flowable and moldable during membrane casting and resulted in uniform membrane formation during the phase inversion process. To investigate the influence of CNT concentration on membrane performance, three 13 wt% PVDF membranes were prepared using CNT concentrations of 0.3, 0.5, and 0.7 wt%, respectively. Due to their unique one-dimensional structure, the incorporation of CNTs was expected to enhance pore connectivity, mechanical strength, and vapor transport. All membranes were tested under the same DCMD conditions and same saline feed solution, and their flux and salt rejection measurements were compared. The results are shown in Table 3.

The membrane with 0.5 wt% CNT exhibited the highest water flux of 11.5 LMH, while maintaining excellent salt rejection. At this concentration, the CNTs were well-dispersed in the polymer matrix, forming a more interconnected pore network that facilitates efficient vapor transport. Moreover, the moderate CNT loading improved hydrophobicity without causing pore blockage.

The 0.3 wt% CNT membrane exhibited the lowest flux among the three membrane formulations. At such a low loading, the CNTs were insufficient to significantly alter the pore structure or enhance vapor transport pathways, resulting in performance similar to that of the pristine PVDF membrane with only limited flux improvement. This suggests that a minimum CNT concentration is necessary to achieve noticeable enhancements. A similar trend was reported by another study, where increasing the CNT content from 0.2% to 0.3% led to an improved flux [62].

The 0.7 wt% CNT membrane showed intermediate performance, with flux higher than that of the 0.3 wt% CNT membrane but lower than that of the 0.5 wt% membrane. This reduced flux is attributed to CNT agglomeration at higher loadings, which partially blocked pores and limited effective vapor transport channels. Although mechanical reinforcement and salt rejection remained satisfactory, the excessive CNT content hindered further flux improvement. Comparing these results with Table 1, the addition of 0.3%, 0.5%, and 0.7% CNT led to an increase in flux of 21.4%, 32.9%, and 27.2%, respectively, relative to the pristine PVDF membrane.

Although a clear trend in flux values was observed, a one-way ANOVA showed limited statistically significant differences, which is likely due to the limited number of replicates. Future work is required to repeat the experiments with a higher number of replicates to improve statistical confidence.

## 4. Conclusions

This study systematically evaluated the effects of polymer concentration and nanofiller incorporation on PVDF membrane performance. Among the formulations, 13 wt% PVDF was identified as optimal, offering improved morphology and overall performance. Comparative testing of CNT, GO, and chitosan showed that CNTs provided the greatest enhancement, with 0.5 wt% CNT yielding uniform dispersion, improved pore structure, enhanced mass transport, and superior separation efficiency. Lower or higher CNT loadings reduced performance, highlighting 0.5 wt% as the optimal nanofiller content for advanced membrane applications. As in many membrane separation processes, temperature polarization and concentration polarization remain the main challenges in membrane distillation. These phenomena reduce the effective driving force across the membrane, which leads to a lower vapor flux and reduced overall efficiency of the process. Future research should focus on developing advanced membrane materials and improving membrane surface properties to reduce temperature and concentration polarization. Carbon-based nanomaterial membranes can help to alleviate these challenges. Specifically, nanomaterials that can improve thermal conductivity, enhance mass transfer, and improve the chemical, mechanical, and thermal stability of the membrane are of high priority. Carbon-based nanofillers are expected to further improve membranes’ distillation performance.

## Figures and Tables

**Table 1:** Membrane flux and rejection using PVDF at different concentrations.

PVDF Conc	Average Flux (LMH)	Standard Error	Average Salt Rejection (%)	Standard Error
10%	9.175	1.025	99.05	0.15
13%	8.65	0.65	99.2	0.2
20%	6.5	0.5	99.9	0.005

**Table 2:** Membrane flux and rejection using 13 wt% PVDF with different additives at 0.5% each.

13 wt% PVDF-Additive	Average Flux (LMH)	Standard Error	Average Salt Rejection (%)	Standard Error
CNT	10.5	0.3	99.6	0.3
GO	10	1	99.45	0.15
CH	9.5	1.5	99.05	0.15

**Table 3:** Membrane flux and rejection using 13 wt% PVDF at different concentrations of CNT.

13% PVDF-CNT	Average Flux (LMH)	Standard Error	Average Salt Rejection (%)	Standard Error
0.3%	10.5	1.5	98.5	0.5
0.5%	11.5	0.5	98.7	0.8
0.7%	11	0.5	98.4	0.9

## Data Availability

The raw data supporting the conclusions of this article will be made available by the authors on request.
